# City-level climate change mitigation in China

**DOI:** 10.1126/sciadv.aaq0390

**Published:** 2018-06-27

**Authors:** Yuli Shan, Dabo Guan, Klaus Hubacek, Bo Zheng, Steven J. Davis, Lichao Jia, Jianghua Liu, Zhu Liu, Neil Fromer, Zhifu Mi, Jing Meng, Xiangzheng Deng, Yuan Li, Jintai Lin, Heike Schroeder, Helga Weisz, Hans Joachim Schellnhuber

**Affiliations:** 1 School of Environment, Tsinghua University, Beijing 100084, China.; 2 Water Security Research Centre, Tyndall Centre for Climate Change Research, School of International Development, University of East Anglia, Norwich NR4 7TJ, UK.; 3 Department of Earth System Science, Tsinghua University, Beijing 100080, China.; 4 Department of Geographical Sciences, University of Maryland, College Park, MD 20742, USA.; 5 Department of Environmental Studies, Masryk University, Joštova 10, 602 00 Brno, Czech Republic.; 6 International Institute for Applied Systems Analysis (IIASA), Schlossplatz 1, A-2361 Laxenburg, Austria.; 7 Laboratoire des Sciences du Climat et de l’Environnement, CEA-CNRS-UVSQ, UMR8212, Gif-sur-Yvette, Paris, France.; 8 Department of Earth System Science, University of California, Irvine, Irvine, CA 92697, USA.; 9 Department of Civil and Environmental Engineering, University of California, Irvine, Irvine, CA 92697, USA.; 10 School of Materials Science and Engineering, State Key Lab of Material Processing and Die and Mould Technology, Huazhong University of Science and Technology, Wuhan, Hubei 430074, China.; 11 Institute of Finance and Economics Research, School of Urban and Regional Science, Shanghai University of Finance and Economics, Shanghai 200433, China.; 12 Resnick Sustainability Institute, California Institute of Technology, Pasadena, CA 911125, USA.; 13 Bartlett School of Construction and Project Management, University College London, London WC1E 7HB, UK.; 14 Department of Politics and International Studies, University of Cambridge, Cambridge CB3 9DT, UK.; 15 Institute of Geographic Sciences and Natural Resources Research, Chinese Academy of Sciences, Beijing 100101, China.; 16 University of Chinese Academy of Sciences, Beijing 100049, China.; 17 College of Economics, Jinan University, Guangzhou 510632, China.; 18 Laboratory for Climate and Ocean-Atmosphere Studies, Department of Atmospheric and Oceanic Sciences, School of Physics, Peking University, Beijing 100871, China.; 19 Potsdam Institute for Climate Impact Research, 14473 Potsdam, Germany.; 20 Department of Cultural History and Theory and Department of Social Sciences, Humboldt University of Berlin, Unter den Linden 6, 10117 Berlin, Germany.; 21 University of Potsdam Stockholm Resilience Centre, Stockholm, Sweden.

## Abstract

As national efforts to reduce CO_2_ emissions intensify, policy-makers need increasingly specific, subnational information about the sources of CO_2_ and the potential reductions and economic implications of different possible policies. This is particularly true in China, a large and economically diverse country that has rapidly industrialized and urbanized and that has pledged under the Paris Agreement that its emissions will peak by 2030. We present new, city-level estimates of CO_2_ emissions for 182 Chinese cities, decomposed into 17 different fossil fuels, 46 socioeconomic sectors, and 7 industrial processes. We find that more affluent cities have systematically lower emissions per unit of gross domestic product (GDP), supported by imports from less affluent, industrial cities located nearby. In turn, clusters of industrial cities are supported by nearby centers of coal or oil extraction. Whereas policies directly targeting manufacturing and electric power infrastructure would drastically undermine the GDP of industrial cities, consumption-based policies might allow emission reductions to be subsidized by those with greater ability to pay. In particular, sector-based analysis of each city suggests that technological improvements could be a practical and effective means of reducing emissions while maintaining growth and the current economic structure and energy system. We explore city-level emission reductions under three scenarios of technological progress to show that substantial reductions (up to 31%) are possible by updating a disproportionately small fraction of existing infrastructure.

## Teaser

Technological advancement in industrializing cities is critical for reducing CO_2_ emissions while maintaining economic growth.

## INTRODUCTION

Under the Paris Agreement, China pledged that its emissions will peak by 2030 and that it will decrease the carbon intensity [CO_2_ emissions per unit of gross domestic product (GDP)] of its economy by 60 to 65% relative to 2005 levels (*1*). To fulfill these ambitious commitments in the most cost-effective way, policy-makers seek to characterize the sources of CO_2_ in as much detail as possible and to assess the potential for emission reductions and economic losses related to different policy approaches.

Urbanization is a major driver of economic growth in China, and—as elsewhere—cities produce most (85%) of China’s CO_2_ emissions (*2*). For this reason, China’s cities play an increasingly important role in its efforts to reduce CO_2_ emissions. For example, the newly launched Emission Trading Scheme in 2017 intends to monitor and control national CO_2_ emissions and energy consumption at the city/firm level as part of an emission peak by 2030. Currently, the Emission Trading Scheme covers only the electricity sector owing to data accessibility. The further success of the scheme will depend on accurate sector-level accounts of city emissions, as well as the cooperation of city-level governments where many officials are concerned about the economic impacts of energy and emission constraints. However, in comparison to national and provincial emissions, data to support city-level emission inventories are generally less available and of lower quality.

Because of these data limitations, previous studies have focused on megacities in developed countries for which energy data were consistent and systematic, particularly cities in the United States. For example, Ramaswami *et al.* (*3*) developed a hybrid life cycle–based methodology for conducting city-scale greenhouse gas inventories, and their subsequent studies (*4*) applied the method to eight U.S. cities. Kennedy *et al.* (*5*, *6*) also estimated the emissions of 10 megacities. Matese *et al.* (*7*) estimated the CO_2_ emissions of Florence, Italy, based on carbon flux observations. Later, attempts were also made to estimate the city-level emissions in some developing countries. D’Almeida Martins and da Costa Ferreira (*8*) analyzed emissions of two Brazil megacities: São Paulo and Rio de Janeiro. Ali and Nitivattananon (*9*) discussed the emissions from the city of Lahore (in Pakistan) from 1971 to 2010.

Only recently have studies begun to include large and rapidly growing Chinese cities (*10*, *11*), usually by adopting statistical downscaling methods. For example, Creutzig *et al.* (*12*) built an energy/emission data set of 274 global cities, assessing their aggregate potential for urban climate change mitigation. Of the 274 cities, 37 are from China. However, the data in Creutzig *et al.* (*12*) were collected from multiple sources of inconsistent statistical caliber and quality. Dhakal (*13*) similarly estimated the emissions of provincial capital cities in China using provincial average energy intensity (energy per GDP energy consumption) and GDP index, thus introducing large uncertainties. In contrast, Wang *et al.* (*14*) analyzed the emissions from China’s provincial capital cities based on the cities’ statistical yearbooks. Ramaswami *et al.* (*15*) further estimated emissions from 637 Chinese cities by downscaling from national/provincial data (*16*).

Yet, each of these previous studies assessed emissions using different methods, scopes, and primary data (see Materials and Methods for details), generating inconsistent results and preventing meaningful comparisons across studies. For example, Parshall *et al.* (*17*) and Markolf *et al.* (*18*) only estimated the scope 1 emissions, whereas Ramaswami *et al.* (*3*) included both direct and indirect emissions and Ramaswami *et al.* (*16*) estimated the scope 1 + scope 2 emissions for cities. The scope 1 emissions refer to CO_2_ emitted during energy combustion or other human activities within a city boundary, while the scope 2 emissions included CO_2_ induced by imported electricity/heat generation. Mi *et al.* (*19*) calculated the consumption-based (scope 3) emissions of 13 Chinese cities using an input-output method. The consumption-based emissions quantify the emissions embodied in the consumption of final products.

Furthermore, most of the previous studies of city-level emissions provided a single number for total emissions (*13*) or, alternatively, included only emissions from selected key sectors. Thus, the sketchy emission accounts of cities cannot support in-depth discussion of city-level emission characteristics and of potential policies for emission reduction. In addition, emission sources reported in different studies are disparate, increasing the uncertainties and discrepancies across studies. For example, Ramaswami *et al.* (*3*) calculated emissions from “buildings and facilities’ energy final consumption, transportation, and embodied energy consumption of key urban materials.” On the other hand, Kennedy *et al.* (*6*) calculated emissions from seven sectors: “electricity, heating and industrial fuels, ground transportation fuels, aviation and marine transportation, industrial processes, product use, and waste.” In contrast, Wang *et al.* (*14*) calculated city-level emissions from “industries, transportation, household energy use, commerce, industrial processes, and waste.”

Comprehensive and consistent inventories of city-level emissions based on physical energy flows are thus still badly needed, including disaggregation of fossil fuel types and socioeconomic sectors within cities’ boundaries. Our study provided the CO_2_ emission inventories for 182 Chinese cities by 17 fossil fuels and 46 socioeconomic sectors. We follow the Intergovernmental Panel on Climate Change (IPCC) administrative territorial approach, which is compatible and consistent with national and international emission inventories. The 46 sectors are classified according to China’s System of National Accounts (further details in Materials and Methods). The sectors classification can also be mapped with other countries/cities around the world (*20*). The result is a set of consistent and directly comparable CO_2_ emission inventories of cities that can provide robust and transparent data support for city-level emission control in China, as well as the nationwide Emission Trading Scheme.

The cities are defined as prefecture-level administrative units in China (including both build-up city and administrative area). According to the latest administrative planning report, there are currently 334 cities in China. We select 182 cities based on the data availability (see Materials and Methods for data source). The 182 case cities encompass 62% of China’s population as well as 77% of its GDP in 2010.

We first classify cities into five groups according to their apparent development pathway and then quantify the potential for emission reductions among the city groups under a range of technological scenarios. Our results reveal the extent to which different policies may reduce emissions while in large part maintaining the current industrial structure and energy mix of cities and thereby minimize economic impacts.

## RESULTS

### Emission inventories of 182 cities

**Fig. 1:**
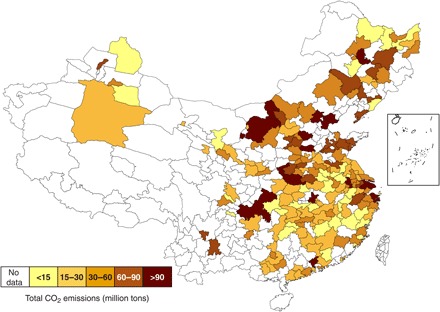
Total CO_2_ emissions of 182 Chinese cities.

Figure 1 shows the total CO_2_ emissions of 182 cities, which are for 2010. Most of the 182 cities are located in the eastern half of the country [that is, under the Aihui-Tengchong line, where more than 90% of China’s population resides (*21*)]. In 2010, the 182 cities emitted 7610 million tons (Mt) of CO_2_ in total. Industrial sectors make up the largest share of emissions (6639 Mt of CO_2_ or 87% of the cities’ total), especially power and heat production, iron and steel production, and nonmetal minerals (cement, glass, and ceramics). Nonindustrial sectors make up the remaining 12% (971 Mt of CO_2_), with two-thirds of emissions from farming and direct energy use in rural areas. The pie charts in fig. S1 (A and B) show the sector mix of 182 cities’ total CO_2_ emissions. Figure S1C shows that burning of coal is the source of 74% [especially raw coal (57%) and coke (9%)] of the cities’ total emissions, with oil and natural gas combustion representing just 15 and 2%, respectively, and the remaining 9% from industrial processes such as cement production.

Population and socioeconomic development varies tremendously among these cities: from 0.2 million people living in Jiayuguan (Gansu province, northwest China) to 28.7 in Chongqing (southwest) and from per-capita GDP of just ¥9068 in Fuyang (Anhui province, east) to ¥175,125 in Ordos (Inner Mongolia, north). Similarly, colors in Fig. 1 indicate that the total CO_2_ emissions of the 182 cities range from <15 Mt (yellow) to >90 Mt (dark red; see also table S1). Higher levels of emissions prevail in the north and east, and the top-emitting cities represent a disproportionately large fraction of the total emissions from the 182 cities. The top five emitting cities [Tangshan, Shanghai, Suzhou (the one in Jiangsu province), Nanyang, and Chongqing] accounted for 11% of the total in 2010. In addition, our estimates of CO_2_ emission intensity, calculated as total emissions divided by GDP, range from <0.1 to >1.5 metric tons of CO_2_ per ¥1000, with a minimum of 0.04 and 0.05 metric tons of CO_2_ per ¥1000 in Shenzhen (Guangdong province, east coast) and Huangshan (Anhui province, east), respectively; a maximum of 1.72 and 1.55 metric tons of CO_2_ per ¥1000 in Hegang (Heilongjiang province, northeast) and Panzhihua (Sichuan province, southwest), respectively; and an average of 0.22 metric tons of CO_2_ per ¥1,000 (see table S1).

The detailed investigation of cities’ emissions by sectors and fuels helps in understanding the wide range in cities’ carbon intensity: cities such as Beijing and Shenzhen, whose emission intensities are just 0.07 and 0.04 metric tons of CO_2_ per ¥1000, respectively, have small manufacturing and energy sectors (20 and 44% of their GDP, respectively) and larger service sectors (75 and 53%, respectively). In contrast, cities such as Maanshan, Tangshan, and Panzhihua have iron and steel production as their “pillar” industries (21, 27, and 31% of their GDP, respectively), with correspondingly high carbon intensities: 0.68, 0.43, and 1.55 metric tons of CO_2_ per ¥1000, respectively. Similarly, in cities where energy production and mining of natural resources are the dominant industries, such as Hegang, emission intensities are especially high (1.72 metric tons of CO_2_ per ¥1000) because the activities are emission-intensive, but such cities produce low value-added energy products (such as cleaned coal, coke, and electricity).

### Five city groups

**Fig. 2:**
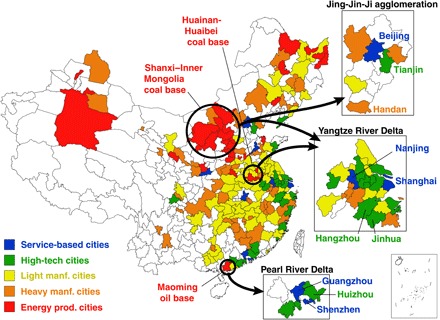
Spatial distribution of five city groups. Colors on the map indicate the categorization of each of the 182 cities into energy production (prod.) cities (red), heavy manufacturing (manf.) cities (orange), light manufacturing cities (yellow), high-tech cities (green), and service-based cities (blue). Black circles and areas indicate the location of coal and oil bases and common city cluster destinations for their energy exports.

Recognizing these characteristic differences, we use formal cluster analysis to classify the cities into five distinct groups based on their GDP and industrial output: service-based cities (*n* = 8), high-tech cities (*n* = 24), energy production cities (*n* = 32), heavy manufacturing cities (*n* = 51), and light manufacturing cities (*n* = 67) (see Materials and Methods). Figure 2 shows the geographical distribution of the cities in each group. Most service-based and high-tech cities (blue and green in Fig. 2, respectively) are located in east and south China, with 21 of the 32 gathered into three city clusters (see map insets of Jing-Jin-Ji, the Yangtze River Delta, and the Pearl River Delta). In contrast, the 32 energy production cities (red in Fig. 2) are congregated in west and north China owing to the location of fossil resources (mainly coal) in those regions. The heavy and light manufacturing cities (orange and yellow in Fig. 2, respectively) are more widely dispersed but with a large concentration in central China.

Yet, none of the different types of cities are independent of the others, and there is evidence of a division of labor among the city groups. For example, although none of the three major city clusters highlighted in Fig. 2 contain cities where energy production is the pillar industry, each cluster is supported by energy imported from nearby energy production centers: The Pearl River Delta cities are supported by Maoming oil base, the Jing-Jin-Ji region obtains power from the Shanxi–Inner Mongolia coal base, and the Yangtze River Delta cities are supported by both the Huainan-Huaibei and Shanxi–Inner Mongolia coal bases (Fig. 2, black circles and arrows, and fig. S2). Emission-intensive activities of outer-lying heavy and light manufacturing cities similarly support production and consumption activities of the major city clusters (*19*, *22*).

**Fig. 3:**
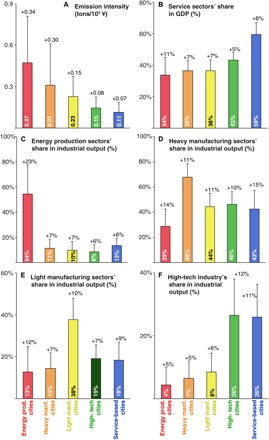
Mean and standard deviation of emission intensity and economic structure of each city group. The bars present the mean value of the variables; the lines above the bars show the +1 SD of the variables.

Figure 3A shows the emission intensity’s mean values and SDs of the five city groups. The average emission intensity is 0.47 metric tons of CO_2_ per ¥1000 in energy production cities, 0.31 metric tons of CO_2_ per ¥1000 in heavy manufacturing cities, 0.23 metric tons of CO_2_ per ¥1000 in light manufacturing cities, 0.15 metric tons of CO_2_ per ¥1000 in high-tech cities, and 0.11 metric tons of CO_2_ per ¥1000 in service-based cities. We find that among the five city groups, the energy production cities have the highest average emission intensity, while the service-based cities have the lowest value. The *z* test of mean values shows that the average emission intensities of the service-based and high-tech cities are significantly lower than those of the energy production and heavy manufacturing cities at the 0.05 level. In addition, the average emission intensity of the light manufacturing cities is significantly lower than that of the energy production cities at the 0.05 level as well (see Materials and Methods). The SDs of the energy production and heavy manufacturing cities’ emission intensity are the largest among the five city groups (0.34 and 0.30 metric tons of CO_2_ per ¥1000, respectively). The SDs of high-tech and service-based cities’ emission intensities are relatively small, which are 0.08 and 0.07 metric tons of CO_2_ per ¥1000, respectively.

Such a difference in city groups’ energy intensity is determined by the cities’ economic structures. The energy production and heavy manufacturing cities have more energy-insensitive sectors, which emit high CO_2_ with low economic outputs. Conversely, high-tech and service-based cities rely more on the high-tech industries and service sectors. This led them to lower emission intensities. Figure 3 (B to F) shows the economic structures of the five city groups.

Figure 3 (B to F) and related *z* test of mean values (see Materials and Methods) define the pillar industry of each city group. A city group’s pillar industry has the highest share in economic structure compared with other city groups. For example, the service sectors’ average share in GDP of the service-based cities (59%) is significantly higher than those of energy production (34%), heavy manufacturing (36%), light manufacturing (36%), and high-tech (43%) cities. Similarly, the energy production sectors’ average share in energy production cities (54%) is the highest among the five city groups.

### Superemitting city sectors and CO_2_ reduction capacities in industrial sectors

Given the strong influence of industrial activities on cities’ CO_2_ emissions, we next examine the potential for emission reductions in industrial sectors through scenarios of specific technological improvement. As each city has 39 industrial sectors (5 energy production, 16 heavy manufacturing, 13 light manufacturing, and 5 high-tech industries), there are 7098 industrial city sectors in total. The term “city sector” refers to each industrial sector of each city; for example, thesector “coal mining and dressing” of Beijing is a city sector, while the sector “food processing” of Shanghai is another. We calculate the per–industrial output emissions for the 7098 industrial city sectors. We then identify three levels of superemitters of those city sectors based on the city sectors’ per–industrial output emissions (*23*).

(1) Above-average emitters have per–industrial output emissions greater than the sector mean.

(2) One SD superemitters have per–industrial output emissions 1σ above the sector mean.

(3) Two SD superemitters have per–industrial output emissions 2σ above the sector mean. The 2 SD superemitters represent the most carbon-intensive city sectors.

**Fig. 4:**
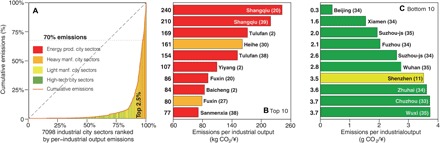
City sectors ranked by per-industrial output emissions. Emission-Lorenz curve of 7098 industrial city sectors (**A**) and top/bottom 10 city sectors in per–industrial output emission (**B** and **C**). The numbers alongside the *y* axis in (B) and (C) are the per–industrial output emissions of the city sectors. The numbers in parentheses after the city name denote the sectors of the cities, which are consistent with the sectors’ ID number in table S2. The colors of the bars indicate the city sectors’ categories (red, energy production; orange, heavy manufacturing; yellow, light manufacturing; green, high-tech industry). For example, Shangqiu (20) in (B) refers to the “petroleum processing and coking” sector of Shangqiu and belongs to energy production (red); Beijing (34) in (C) refers to the “electronic and telecommunications equipment” sector of Beijing and belongs to the high-tech industry (green).

The few superemitters of city sectors represent a disproportionately large fraction of the total emissions. The top 2.5% of the 7098 industrial city sectors in per–industrial output emissions contribute 70% of total CO_2_ emissions (Fig. 4A, emission-Lorenz curve). Figure 4 (B and C) shows the top 10 and bottom 10 industrial city sectors, respectively, in terms of emissions per industrial output.

To explore the potential emission reduction capacities via applying technical improvement to the three levels of superemitting city sectors, we define three scenarios. Scenario #3 is the strongest scenario when all the above-average emitters improve their technology to the average level, while scenario #1 is the mildest scenario.

(1) Scenario #1: 2 SD superemitting city sectors reach the current national sector average emission intensity.

(2) Scenario #2: 1 SD superemitters reach the current sector average.

(3) Scenario #3: Above-average superemitters reach the current sector average.

We then calculate the emission reduction capacities of the five city groups’ pillar industry, under the three scenarios (shown in fig. S3A). Under the strongest scenario, #3, reductions to cities’ pillar industries alone could avoid emissions of 544 Mt of CO_2_ in energy production cities, 388 Mt of CO_2_ in heavy manufacturing cities, 36 Mt of CO_2_ in light manufacturing cities, and 1 Mt of CO_2_ in high-tech cities, or 52, 38, 40, and 11%, respectively, of those cities’ pillar industry emissions. Energy production and heavy manufacturing sectors are the primary emission sources of every city group due to their high emission intensities; therefore, we calculate the emission reduction capacities of the cities’ energy production (fig. S3B) and heavy manufacturing sectors (fig. S3C) as well.

**Fig. 5:**
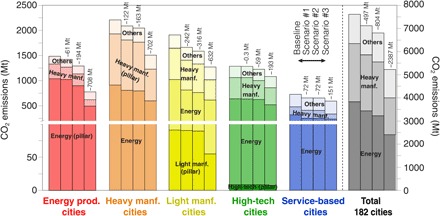
CO_2_ emissions of city groups under three reduction scenarios. Potential reductions in CO_2_ emissions (in million tons) are shown for each of the five city groups where the emission intensities of 2 SD, 1 SD, and above-average superemitters are brought down to the sector mean intensity (scenarios #1, #2, and #3, respectively). The numbers on top of each scenario bar represent the potential reductions in CO_2_ emissions under the scenarios compared with the baseline. The magnitude of reductions under scenario #1 is greatest in the light manufacturing cities, while the energy production cities have the largest reduction magnitude under scenario #3. The overall reductions under scenario #3 are 2.4 Gt of CO_2_, or 31% of the cities’ emissions in 2010.

Figure 5 and Table 1 summarize comprehensive results of the potential emission reductions of city groups’ pillar industry, energy production, and heavy manufacturing sectors. We presented the results under three scenarios relative to a baseline of current emissions.

**Table 1:** CO_2_ emissions of five city groups by sector categories under the three reduction scenarios (2010, million tons).

***City groups***	***Scenario***	***Energy production***	***Heavy manufacturing***	***Light manufacturing***	***High-tech***	***Farming, construction, services, and household***	***Total***
Energy production cities	Baseline	1037.26	291.28	28.16	4.71	123.97	1485.37
	Scenario #1	1022.23	245.35	28.16	4.71	123.97	1424.42
	Scenario #2	897.47	236.99	28.16	4.71	123.97	1291.30
	Scenario #3	493.63	126.42	28.16	4.71	123.97	776.89
Heavy manufacturing cities	Baseline	912.60	1017.10	48.24	4.76	225.90	2208.61
	Scenario #1	808.38	999.65	48.24	4.76	225.90	2086.94
	Scenario #2	796.62	970.20	48.24	4.76	225.90	2045.73
	Scenario #3	599.36	628.84	48.24	4.76	225.90	1507.11
Light manufacturing cities	Baseline	928.88	629.15	90.87	7.22	246.99	1903.10
	Scenario #1	723.50	592.64	90.34	7.22	246.99	1660.68
	Scenario #2	670.52	572.87	89.37	7.22	246.99	1586.97
	Scenario #3	562.16	399.96	54.86	7.22	246.99	1271.18
High-tech industry cities	Baseline	607.05	393.85	53.46	8.28	157.55	1220.18
	Scenario #1	607.00	393.57	53.46	8.27	157.55	1219.84
	Scenario #2	591.56	350.45	53.46	8.27	157.55	1161.29
	Scenario #3	489.64	318.66	53.46	7.39	157.55	1026.68
Service-based cities	Baseline	341.79	190.15	23.02	20.94	216.45	792.36
	Scenario #1	270.51	189.75	23.02	20.94	216.45	720.68
	Scenario #2	270.51	189.55	23.02	20.94	216.45	720.48
	Scenario #3	256.67	123.91	23.02	20.94	216.45	640.99
Total (all 182 cities)	Baseline	3827.57	2521.53	243.75	45.90	970.86	7609.62
	Scenario #1	3431.63	2420.95	243.22	45.90	970.86	7112.56
	Scenario #2	3226.69	2320.05	242.26	45.90	970.86	6805.76
	Scenario #3	2401.45	1597.79	207.74	45.01	970.86	5222.86

Although statistically few, mitigating emissions from only 2 SD superemitters could nonetheless substantially reduce CO_2_ emissions, particularly in heavy manufacturing and light manufacturing cities (the second bars of each city group in Fig. 5). The emissions reduced under scenario #1 of the energy production, heavy manufacturing, light manufacturing, high-tech manufacturing, and service-based cities are 61 Mt (or 4%), 122 Mt (6%), 242 Mt (13%), 0.3 Mt (0.03%), and 72 Mt (9%), respectively. If scenario #3 technical improvement is applied to cities’ pillar, energy production, and heavy manufacturing industries, then the overall reductions are 2.4 gigatons (Gt) of CO_2_, or 31% of the cities’ emissions in 2010 (shown in Fig. 5 and Table 1).

## DISCUSSION

Updating and improving technologies might reduce emissions while leaving the industrial structure of individual cities (and thus their respective roles in the existing Chinese economy) unchanged. This is critical, given the rapidity of targeted reductions; the central government in China seeks to reduce national CO_2_ emission intensity by 60 to 65% compared with the 2005 level by 2020 (*1*). If the percentage reductions of scenario #3 in 2010 (31%) are assumed to still be available in 2014, then China’s emission intensity in 2014 could be 48.25% lower than the 2005 level (the real emission intensity in 2014 is 25% lower than the 2005 level). This would allow China to meet its 60 to 65% commitments easier. Other options for meeting these goals, such as radical industrial restructuring or large-scale shifts in the country’s energy mix (*24*), are unlikely to be feasible over the time span of just a few years. For example, policies that simply shut down energy-intensive industries would immediately and seriously affect the economies of energy production and heavy industry cities, as well as the more developed high-tech and service-based cities that depend on them.

Although it would be costly and disruptive for energy production– and heavy industry–based cities to reorganize their industrial structure in the short term (for example, by closing or relocating emission-intensive industries), more affluent, service-based cities might be able to quickly outsource their more emission-intensive industries without economic hardship. However, such outsourcing by highly developed cities could increase overall emissions; by virtue of their level of development, these cities often have more advanced technologies in place, and outsourcing would thus tend to move carbon-intensive, heavy-polluting industries to less-developed regions with less-efficient technologies. For example, Shougang Corporation, one of the largest steelmaking companies in China, has moved progressively from Beijing to Hebei province (mainly to Tangshan) since 2010. Beijing’s CO_2_ emissions decreased by 7.6 Mt during 2010 to 2015, but emissions in Hebei province increased by 87.1 Mt during the same period (*25*), and Shougang Corporation is one of the main causes of the increase. Whereas the emission intensity of iron and steel production in Beijing was 1.4 metric tons of CO_2_ per ¥1000 in 2007, the intensity of the same sector in Tangshan was 2.6 metric tons of CO_2_ per ¥1000 in 2010 (86% higher). Although a few affluent cities have reduced the proportion of coal in their energy mix [for example, Beijing has reduced its coal consumption by 61%, or 18.2 Mt from 2007 to 2015 (*26*, *27*)] through a combination of increased renewables and natural gas, China’s large stocks of cheap coal and equally large fleet of young, coal-burning power plants (*28*) are daunting economic barriers to radical near-term shifts in the Chinese energy mix.

On the basis of detailed analysis of cities and their industries, our findings suggest that China’s near-term goals of reducing its emission intensity may be feasibly accomplished by targeted technological improvements, buying time for the longer-term strategies of shifting to non-fossil energy and a more service-based economy. Moreover, improving and optimizing the energy and carbon efficiency of industrial production processes and operations could help lower the costs of advanced technologies and thus facilitate their deployment in less-developed cities and countries beyond China.

## CONCLUSION

In order for China to cost-effectively reach its goal of reducing CO_2_ emission intensity by 60 to 65% and running a nationwide Emission Trading Scheme over the next few years, policy-makers in the country need increasingly specific, subnational information about the sources of CO_2_ and the potential reductions and economic implications of different possible policies. By categorizing Chinese cities according to their development stage and industrial makeup, we offer policy-makers an opportunity to meaningfully differentiate across the wide range in city-level CO_2_ emissions (from 1.6 to 194 Mt) and emission intensity (from 0.04 to 1.72 metric tons of CO_2_ per ¥1000). Further, because the lower emission intensities of affluent cities (that is, high-tech and service-based cities) are supported by imports from less affluent, industrial, and energy-producing cities located nearby, consumption-based policies may allow more developed cities to subsidize emission reductions without undercutting the economic core of still-developing cities by directly regulating their manufacturing and the electric sectors.

However, where policies directly targeting manufacturing and electric power infrastructure are implemented, our sectoral analysis of each city indicates that targeted technological improvements may be a practical and effective means of reducing emissions while preserving cities’ current economic structure and energy systems. In particular, by focusing efforts on superemitting industry sectors in each city, roughly 30% of China’s carbon emissions might be eliminated.

Although the leveling off of China’s CO_2_ emissions in recent years is a tremendous watershed in the global effort to avoid dangerous climate change, the progress reflects sweeping policies to improve the country’s industrial technologies and energy systems. Further progress will increasingly depend on policies that differentiate among cities according to their economic structure, level of development, and infrastructure and are carefully crafted to target the largest and most cost-effective emission reductions.

In addition, rapidly growing cities with “new” investments in infrastructure and institutions tend to emulate already-established cities and thereby lock into the “old” development paths. Instead, promoting low-carbon transitions at early stages of industrialization implies careful consideration of “future fit”—how industries can be designed and run to optimize or decouple the relationship between their economic productivity and energy use (or environmental impact). The socioeconomic and environmental characteristics of the 182 cities represent various stages of industrialization, from the preindustrialization stage (for example, energy production cities in Shanxi and Inner Mongolia) to the postindustrialization stage (such as Beijing and Shanghai). In turn, this cross section of Chinese cities we analyze may provide important insights for other developing countries seeking to target superemitting sectors/units in their cities, perhaps enabling them to bypass or abbreviate the most emission-intensive phases of industrialization.

## MATERIALS AND METHODS

### CO_2_ emission accounts and data source

#### Scopes

Three approaches are usually used to account for the CO_2_ emissions of one country: the territorial-based, production-based, and consumption-based approaches. According to the IPCC (*29*), the territorial-based emissions are CO_2_ emitted within one administrative unit. In the production-approach, not only “emissions from international aviation and shipping are typically allocated to the country of the relevant vessel’s operator” [(*30*), p. 453] but also the “emissions from international tourism are allocated based on where individual tourists are resident, rather than their destination” [(*30*), p. 453]. The consumption-based emissions are calculated according to the final products’ consumption (*31*).

Similarly, three “scopes” are defined by the World Resources Institute (WRI) and the World Business Council for Sustainable Development (*32*) to account for the regional CO_2_ emissions. Scope 1 includes emissions from in-boundary fossil fuel combustion, industrial process/product use, wastes, and other in-boundary activities. Scope 2 refers to the in-boundary electricity/heat-related emissions induced by the purchased electricity and heat. Scope 3 includes all the out-of-boundary emissions such as emissions from aviation/marine and imported products/services (*6*). Accordingly, four system boundaries for regional emission accounts are defined on the basis of the three scopes: system boundary 1 is equal to scope 1 emissions; system boundary 2 includes both scope 1 and scope 2 emissions; system boundary 3 is equal to scope 1 plus scope 3 emissions; while system boundary 4 is consumption-based emissions (also called carbon footprint) (*33*).

Considering the higher uncertainties and incomparability of the scope 3 emissions, most of the previous studies on city-level emission accounts focus mainly on the scope 1 territorial emissions (*34*). Here, we considered the scope 1 territorial emission as well owing to the city-level data accessibility in China. The territorial emissions describe the current CO_2_ emission induced within one country/region’s administrative boundary. The territorial emissions are usually used for the emission feature analysis and for the reduction policy-making. Territorial emissions are the foundation of the other emission approaches, in that production- and consumption-based emissions are calculated on the basis of territorial emissions.

The territorial CO_2_ emissions of cities in this study included both the fossil fuel–related and process-related emissions. The fossil fuel–related emissions were induced by the fossil fuel combustions. Per the definition of territorial direct CO_2_ emissions, the cities’ CO_2_ emissions in this study did not include the part induced by imported/purchased electricity and heat. The emissions from the fossil fuel burnt in power plants for electricity/heat generation were allocated to the power and heat sector of the location cities. In addition, we removed the energy used as raw materials during industrial processes (shown as non-energy use in energy statistics) from the total consumption; this part did not emit CO_2_ either (*35*). The process-related emissions referred to CO_2_ that escaped from chemical reaction during the industrial processes (*36*), rather than emissions from fossil fuel combustion to gain power, which were accounted as fossil fuel–related emissions. Above all, this study calculated the cities’ IPCC territorial administrative emissions from 17 kinds of fossil fuels (fossil fuel–related; see table S3) and 7 industrial processes (process-related; see table S4).

#### CO_2_ emission calculation method

IPCC (*29*) provided methods for CO_2_ emission accounting based on mass-balance theory. The emissions were estimated as the product of activity data (energy consumption or industrial productions) and the corresponding emission factors. The method is widely used by researchers. On the basis of the IPCC method, the National Development and Reform Commission of China (NDRC) designed an emission account system for Chinese provinces (*35*). Furthermore, in the “Global Protocol for Community-Scale Greenhouse Gas Emission Inventories” and “International Local Government GHG Emissions Analysis Protocol”, WRI *et al.* (*37*) and Local Governments for Sustainability (ICLEI) (*38*) provided a bottom-up approach for higher-precision city-level emission accounting. The ISO 14064 and 37120 series of standards also provided guidelines for emission accounts at the enterprise level (*39*).

Alternative approaches were developed to account for the city-level emissions. For example, Cai *et al.* (*40*) used spatial models to build a bottom-up emission database for Chinese cities at 1 km × 1 km resolution. Doll *et al.* (*41*), Ma *et al.* (*42*), and Meng *et al.* (*43*) used nighttime light imagery to estimate city CO_2_ emissions. These approaches can potentially provide more detailed emissions or full-coverage emissions for Chinese cities. However, they have higher data requirements compared with the IPCC methods, making them difficult to implement. Furthermore, city-level emissions calculated by these methods are not comparable with the national emission inventories due to differences in methods and scopes.

Here, we adopted the IPCC and NDRC methods. The CO_2_ emissions from fossil fuel combustion (CE_energy_) were calculated in Eq. 1, while the process-related emissions (CE_process_) were calculated in Eq. 2.

$${\text{CE}}_{\text{energy}}=\sum_{i}\sum_{j}{\text{CE}}_{ij}=\sum_{i}\sum_{j}{\text{AD}}_{ij}\times {\text{NCV}}_{i}\times {\text{CC}}_{i}\times O_{ij}$$ (1)

In Eq. 1, CE_*ij*_ refers to the CO_2_ emissions by fossil fuel type *i* and sector *j*; AD_*ij*_, the activity data, means the corresponding fossil fuel consumption. NCV_*i*_, CC_*i*_, and *O*_*ij*_ are known as emission factors. NCV_*i*_ refers to net caloric value, which is the heat value produced per physical unit of fossil fuel combusted (in J/ton); CC_*i*_ (carbon content) is the CO_2_ emissions per net caloric value produced from a given fossil fuel type *i* (in metric tons of CO_2_/J); *O*_*ij*_ is the oxygenation efficiency, which refers to the oxidation ratio when fossil fuels are burned (in %).

NCV_*i*_ and CC_*i*_ were collected on the basis of our previous investigations on China’s fossil fuel quality (*44*), which were assumed to be more suitable for China, and are now being used by the Chinese government in its recently released report on climate change (see table S3) (*45*). *O*_*ij*_ values were collected from NDRC (*35*), which were considered differently for fossil fuels used in different sectors, as the combustion technology level of sectors are different in China. The discussion of emission factors’ comparisons and detailed data are presented in our previous study (*25*).

In Eq. 2, CE_process_ refers to the CO_2_ emissions emitted from industrial processes. AD_*t*_ refers to the production of industrial process *t*, while EF_*t*_ is the corresponding emission factor. The emission factors (EF_*t*_) were collected from IPCC (*29*) and NDRC (*35*) (see table S4).

$${\text{CE}}_{\text{process}}=\sum_{t}{\text{CE}}_{t}=\sum_{t}{\text{AD}}_{t}\times {\text{EF}}_{t}$$ (2)

#### Emission inventory construction and formatting

As discussed above, our direct territorial CO_2_ emissions included 17 kinds of fossil fuels (*i* ∈ [1, 17]) and 7 industrial processes (*t* ∈ [1, 7]). The fossil fuel–related emissions were calculated on the basis of 46-sectoral energy consumption; therefore, the cities’ emission inventories were constructed by 46 socioeconomic sectors (*j* ∈ [1, 46]), corresponding to the national sectors classification defined by the National Administration for Quality Supervision and Inspection and Quarantine (see table S2) (*46*). The sector classification is widely used in the System of National Accounts in China.

The 46 sectors include 1 farming, 1 construction, 3 service sectors, 2 household energy use (urban and rural), and 39 industrial sectors. The 39 industrial sectors can be grouped into four categories: energy production, heavy manufacturing, light manufacturing, and high-tech industries. The energy production category includes five sectors that produce either primary or secondary energy types, while the high-tech category refers to five sectors aiming at high and new technical industries. The remaining 29 sectors belong to manufacturing sectors, including both heavy and light manufacturing sectors. Here, we defined the heavy manufacturing category to include 16 sectors that input energy to produce intermediate products, such as “ferrous metals mining and dressing” and “nonmetal mineral products.” We classified 13 sectors as light manufacturing sectors, which mainly produce final products such as food processing and furniture manufacturing. We allocated the CO_2_ emissions from seven industrial processes to the following sectors: “raw chemical materials and chemical products,” “nonmetal mineral products,” and “smelting and processing of ferrous metals” (see table S4).

#### Activity data collection and source

The 46-sectoral fossil fuel consumptions (AD_*ij*_) were collected on the basis of the “energy balance table” and “industrial sectoral energy consumption table” from the city’s statistical yearbook. We collected the energy consumption data of the nonindustrial sectors (that is, farming, service sectors, and household energy use) from the energy balance table. The energy balance table illustrates the energy data of one region, such as production, import, input and output transformation, final consumption, and loss (see table S5). Because of the poor data quality at the city level, only 10% (or 17) of the 182 cities have the energy balance table in their statistical yearbooks. The remaining 90% (or 165) of the cities do not have the table. Considering the completeness and consistency of cities’ emission inventories, we followed our previous study to scale down the corresponding provincial tables to obtain the city-level energy consumption data of these nonindustrial sectors (*47*). We used the GDP for the farming, construction, and service sectors, assuming that the city has the same farming/construction/service energy intensities as its province, and used the urban/rural population for the urban/rural household energy consumption, assuming that the city has the same per-capita residential energy consumption as its province. The data of GDP and population were collected from cities’ and their corresponding provinces’ statistical yearbooks.

As for the energy consumption data from the 39 industrial sectors, the energy balance table only provides the total amount of the 39 sectors (shown as one industry sector). Here, we collected the energy consumption of the cities’ 39 industrial sectors from the industrial sectoral energy consumption table, which were investigated by the cities’ statistics office at the enterprise level.

The industrial productions of cities (AD_*t*_) were collected from cities’ statistical yearbooks directly. Cities’ population, GDP, industry output, and other socioeconomic data were collected from cities’ statistical yearbooks and *China City Statistical Yearbook 2011*. Results and original data were freely available for download from China Emission Accounts and Datasets (CEADs; www.ceads.net) after registration.

#### Consistency with the national and provincial inventories in China

The city-level emission inventories in this study were constructed by the territorial scope, IPCC calculation method, and framework that were consistently used in China’s official emission accounts and our previous studies of the national and provincial level. Such consistency makes the multiscale emission inventories comparable. The consistent, transparent, comparable emission inventories at multiscales provide robust data support for China’s emission control and the emission trading scheme.

First, the cities’ CO_2_ emissions in this study had the same scope and framework as China’s national and provincial territorial emissions. We included the CO_2_ emissions from the fossil fuel combustion and industrial processes only. The emissions from the electricity and heat generation (which are non-fossil fuels) were allocated to the power plants, rather than to their final consumption sectors. The scope and framework were consistent with the NDRC guidelines for provincial greenhouse gas inventories, which were widely used in China’s emission accounts and studies (*25*, *35*). Second, our cities’ CO_2_ emissions were calculated using the IPCC equation, in which the emissions were equal to activity data times by emission factors. We used China’s most up-to-date emission factors to calculate the cities’ emissions (*44*). The emission factors were also used in the official emission accounts in China (*45*). Moreover, our city-level emission inventories were constructed as 17 fossil fuels and 46 socioeconomic sectors. The 17 fossil fuels were consistent with China’s latest energy statistical system, and the 46 socioeconomic sectors corresponded with the System of National Accounts in China.

The self-consistent emission accounting framework used in this study provides reference for other developing countries without integrated emission accounts. Despite the quality of emission data having been formally required by the “Paris Agreement,” the capacities of inventorying emissions in developing countries remain insufficient, especially in Asia and Africa. Among the 37 developing countries in Asia, only 17 have relatively high inventorying capacities (*48*). The other 20 Asian countries, such as Yemen, North Korea, Kazakhstan, Laos, and Cambodia, have limited inventorying capacities. This is mainly caused by their poor statistical system (*49*). These developing countries with similar limited access to data could refer to this study’s methods for dealing with lack of data to calculate their own CO_2_ emissions.

### Uncertainties of cities’ CO_2_ emission inventories

Emission estimates are subject to uncertainty due to incomplete knowledge of activity data and emission factors. Different methods could also result in potential biases in estimates. To more completely assess uncertainties in our city-level emission accounts, we used the Monte Carlo method recommended by IPCC (*29*) and used by previous scholars widely (*50*). The term “uncertainty” in this study refers to the lower and upper bounds of a certain confidence interval (CI) around our central estimate. All of the input parameters of activity data and emission factors, with assumed normal distributions, were placed in a Monte Carlo framework. Twenty thousand simulations were performed to analyze the uncertainty of estimated emissions by sectors. We assumed that both activity data and emission factors were normally distributed. Coefficients of variation (CVs; SD divided by the mean) were collected from literature: The activity data had CVs ranging from 5 to 30% depending on the sector (*29*, *50*–*54*); the emission factors had CVs of 3% (coal), 1% (oil), and 2% (natural gas) (*44*).

The results show that 97.5% uncertainties (±47.5% CI around the central estimate) of 182 cities fall in (−3.65%, 3.67%). The highest uncertainty appeared in Hegang (−5.83%, 5.86%), while the lowest appeared in Huizhou (−0.91%, 0.91%). Energy production and high emission cities usually have a relatively higher uncertainty of CO_2_ emissions.

In our previous research, we compared our estimation of five cities’ emission with the calculation through a bottom-up approach (*55*, *56*). The result shows that the difference between two results is within 10% (*47*), which is within the uncertainty range and is acceptable for developing countries (*57*, *58*).

### Cluster analysis for city classification

Different methods were used to cluster Chinese cities to discuss previous city-level emissions. For example, Ramaswami *et al.* (*15*) used GDP share to classify 285 Chinese cities into 38 industrial cities, 44 commercial cities, and 203 mixed-economy cities. Such a taxonomy method is further developed based on Nelson (*59*) and is widely used in city-level research.

Here, we combined the cluster analysis and GDP share method to group the 182 cities. The advantage of cluster analysis is that it groups samples with a set of indicators rather than a single one (*60*). Cluster analysis has been widely used in econometrics (*61*, *62*) and other interdisciplinary studies (*63*). The basic rationale of cluster analysis is grouping samples with similar attributes. The samples within the groups will be close together geometrically, while the statistical distance between groups will be farther. The statistical distance is measured by distance metrics, such as Euclidean distance, Manhattan distance, and Minkowski distance.

*K*-means algorithm is one of the cluster algorithms in which the desired number of clusters could be specified in advance before choosing the “best” solution (*64*). According to a previous study on comparison of different distance metrics used in *K*-means algorithm, the Euclidian distance metric’s performance is better than others (*65*). Therefore, we used *K*-means algorithm implemented using the Euclidean distance metric to group cities in this study. The SPSS software was used.

As this study focused on the city-level industrialization process, we used the cities’ manufacturing structure as indicators to conduct the cluster analysis to expose the cities’ pillar industries as well as their position in the industrialization process. The manufacturing structures were calculated on the basis of each city’s sectoral industrial outputs of the four categories’ manufacturing sectors (energy production, heavy manufacturing, light manufacturing, and high-tech industry) in the following steps:

(1) We calculated each city’s industrial output share (IO) of the four manufacturing categories: energy production (IO_EP_), heavy manufacturing (IO_HM_), light manufacturing (IO_LM_), and high-tech industry (IO_HT_). Taking Beijing as an example, IO_EP−Beijing_ is equal to the energy productions’ industrial outputs divided by Beijing’s total industrial outputs (28%). IO_HM−Beijing_, IO_LM−Beijing_, and IO_HT−Beijing_ are 37, 11, and 24, respectively, in 2010. This means 28% of Beijing’s industrial outputs are contributed by energy productions, while 24% are contributed by high-tech industries.

(2) We then sorted the cities according to their sectors’ industrial output share from small to large and obtained the cities’ rankings of each manufacturing category. Taking Beijing as an example, IO_HT−Beijing_ ranks 124 of the total 182 case cities, while IO_HT−Beijing_ ranks 52, which implies that Beijing has more high-tech than heavy manufacturing industries compared with other case cities.

(3) We changed the rankings into percentiles (IO′). Beijing’s high-tech industry share is ahead of 90.11% of the case cities (${\text{IO}}_{\text{HT}-\text{Beijing}}^{\prime }=\frac{124}{182}\times 100\%$).

In this way, we first clustered the 182 case cities into four groups using each city’s manufacturing structure. The cluster analysis results show that group 1 included 22 cities with more energy production enterprises, group 2 included 41 cities with higher ranking in heavy manufacturing, and group 3 included 65 cities that relied more on light manufacturing. The remaining 54 cities were grouped together in group 4 (high-tech).

As the cluster analysis was operated on the basis of mathematical relationships, the results may be distinct with the practical situation. We adjusted the results slightly according to other information and the actual situations in this empirical analysis. For example, Xianyang was moved from group 4 to the energy group due to its high ranking in the energy sector category (88) and huge absolute value of energy production output (¥50,398 million).

Following the cities’ industrialization process, the most developed high-tech cities will further develop into service-based cities. These service cities may go through every stage of the industrialization process in the past decades, and they have now successfully transferred their pillar industry to service sectors with low emission intensity. They presented sophisticated and practical roadmaps of economic structure optimization and low-carbon development. Therefore, this study extracted these service-based cities from the previous high-tech group to form a new group. Here, the service-based cities were defined as high-tech cities with a higher than 50.63% service sectors’ share in GDP. The 50.63% boundary was calculated as the high-tech cities’ average service sectors’ share in GDP (*15*). There were eight cities in the service-based city group: Beijing, Shanghai, Nanjing, Jinan, Wuhan, Guangzhou, Shenzhen, and Xi’an.

**Fig. 6:**
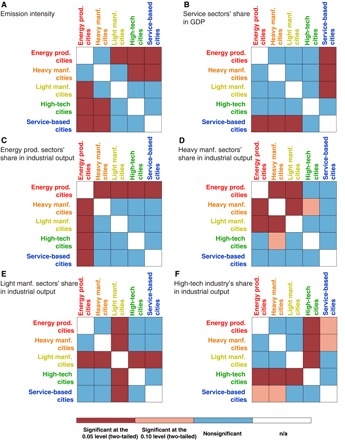
Mean test (*z* test) results of the five city groups. n/a, not applicable.

In this way, the 182 case cities were finally clustered into five city groups with different pillar industries and development pathways. We named the city groups after their pillar industries: 32 energy cities, 51 heavy manufacturing cities, 67 light manufacturing cities, 24 high-tech cities, and 8 service-based cities (see table S1). To validate the city group, we applied the *z* test to compare each city group’s average emission intensity and economic structures, as shown in Fig. 6 and Table 2.

**Table 2:** Mean test (*z* test) results of the five city groups. The *Z* critical one-tailed value is 1.645, while the *Z* critical two-tailed value is 1.960.

***Index***	***City groups***	***z***	***P(Z ≤z) one-tailed***	***P(Z ≤ z) two-tailed***
CO_2_ emission intensity	Service and high-tech cities	−0.221	0.413	0.825
	Service and light manufacturing cites	−0.951	0.171	0.341
	Service and heavy manufacturing cities	−2.033	0.021*	0.042*
	Service and energy production cities	−3.137	0.001*	0.002*
	High-tech and light manufacturing cities	−1.013	0.156	0.311
	High-tech and heavy manufacturing cities	−2.31	0.010*	0.021*
	High-tech and energy production cities	−3.495	0.000*	0.000*
	Light and heavy manufacturing cities	−1.631	0.051^**^	0.103
	Light manufacturing and energy production cities	−2.982	0.001*	0.003*
	Heavy manufacturing and energy production cities	−1.481	0.069^**^	0.139
Service sectors’ share in GDP	Service and high-tech cities	1.435	0.076^**^	0.151
	Service and light manufacturing cites	2.063	0.020*	0.039*
	Service and heavy manufacturing cities	2.177	0.015*	0.029*
	Service and energy production cities	2.107	0.018*	0.035*
	High-tech and light manufacturing cities	1.049	0.147	0.294
	High-tech and heavy manufacturing cities	1.258	0.104	0.208
	High-tech and energy production cities	1.165	0.122	0.244
	Light and heavy manufacturing cities	0.299	0.382	0.765
	Light manufacturing and energy production cities	0.425	0.335	0.671
	Heavy manufacturing and energy production cities	0.205	0.419	0.838
Energy production sectors’ share in industrial output	Service and high-tech cities	0.585	0.279	0.558
	Service and light manufacturing cites	0.304	0.381	0.761
	Service and heavy manufacturing cities	0.287	0.387	0.774
	Service and energy production cities	−3.529	0.000*	0.000*
	High-tech and light manufacturing cities	−0.505	0.307	0.614
	High-tech and heavy manufacturing cities	−0.499	0.309	0.618
	High-tech and energy production cities	−4.846	0.000*	0.000*
	Light and heavy manufacturing cities	−0.021	0.492	0.983
	Light manufacturing and energy production cities	−4.921	0.000*	0.000*
	Heavy manufacturing and energy production cities	−4.816	0.000*	0.000*
Heavy manufacturing sectors’ share in industrial output	Service and high-tech cities	0.139	0.445	0.889
	Service and light manufacturing cites	0.397	0.346	0.691
	Service and heavy manufacturing cities	−1.593	0.056^**^	0.111
	Service and energy production cities	1.410	0.079^**^	0.158
	High-tech and light manufacturing cities	0.160	0.437	0.873
	High-tech and heavy manufacturing cities	−1.877	0.030*	0.061^**^
	High-tech and energy production cities	1.631	0.051^**^	0.103
	Light and heavy manufacturing cities	−4.678	0.000*	0.000*
	Light manufacturing and energy production cities	2.034	0.021*	0.042*
	Heavy manufacturing and energy production cities	5.516	0.000*	0.000*
Light manufacturing sectors’ share in industrial output	Service and high-tech cities	−0.283	0.388	0.777
	Service and light manufacturing cites	−2.068	0.019*	0.039*
	Service and heavy manufacturing cities	0.312	0.378	0.755
	Service and energy production cities	0.196	0.422	0.845
	High-tech and light manufacturing cities	−2.992	0.001*	0.003*
	High-tech and heavy manufacturing cities	1.037	0.150	0.300
	High-tech and energy production cities	0.700	0.242	0.484
	Light and heavy manufacturing cities	4.825	0.000*	0.000*
	Light manufacturing and energy production cities	3.473	0.000*	0.001*
	Heavy manufacturing and energy production cities	−0.150	0.440	0.881
High-tech industry’s share in industrial output	Service and high-tech cities	1.463	0.072^**^	0.144
	Service and light manufacturing cites	1.681	0.046*	0.090^**^
	Service and heavy manufacturing cities	1.801	0.036*	0.072^**^
	Service and energy production cities	2.481	0.007*	0.013*
	High-tech and light manufacturing cities	2.481	0.007*	0.013*
	High-tech and heavy manufacturing cities	2.830	0.002*	0.005*
	High-tech and energy production cities	0.505	0.307	0.613
	Light and heavy manufacturing cities	0.784	0.216	0.433
	Light manufacturing and energy production cities	0.341	0.366	0.733
	Heavy manufacturing and energy production cities	1.463	0.072^**^	0.144

*Significant at the 0.05 level.

^**^Significant at the 0.10 level.

Figure 6A shows that the emission intensity of energy production cities was significantly higher than that of the light manufacturing, high-tech, and service-based cities at the 0.05 level, while the heavy manufacturing cities had a higher average emission intensity than high-tech and service-based cities. The average emission intensities of light manufacturing, high-tech, and service-based cities did not differ significantly. This was determined by the cities’ pillar industry and economic structures. Energy production and heavy manufacturing cities had more energy-intensive enterprises. On the contrary, the high-tech and service-based cities relied more on high-tech industries and service sectors. This can be verified in Fig. 6 (B to F).

In Fig. 6B, the service sectors’ average share in GDP of the service-based cities was significantly higher than those of the energy production, heavy manufacturing, and light manufacturing cities. The service sectors’ average share in GDP of other city groups did not differ significantly. Similar results can be found in the *z* test of energy production sectors (Fig. 6C), light manufacturing sectors (Fig. 6E), and high-tech industries (Fig. 6F). As for the *z* test of the heavy manufacturing sectors, we found that the sectors’ average share in economic structure was significantly different from those of energy production, light manufacturing, and high-tech cities (at the 0.10 level). Moreover, the sectors’ average share was also significantly different between the energy production and light manufacturing cities.

Above all, the *z* test for the mean value between city groups verified the city clusters. The pillar industry of a city group was significantly higher than the others.

### Limitations and future work

This study has some limitations, which mainly lie in the accuracy of the cities’ emission accounts. Further work should focus on the following three aspects to improve the accuracy of the cities’ emission accounts, as well as their emission reduction potentials.

First of all, the downscale emission inventories developed in this study should be compared with the bottom-up emission inventories. As discussed earlier, the bottom-up emissions (*16*, *40*, *42*, *43*) were calculated on the basis of survey data from enterprises. For each city, we would be able to identify a number of key emission-intensive enterprises. Survey data from those enterprises would provide more accurate estimates for those superemitting sectors.

Second, this study developed only 1-year cross-sectional data to define the cities’ pillar industries, which could not disclose the dynamic changes of the cities’ pillar industries, that is, the historical development pathways. In the future, time-series data should be developed to illustrate the cities’ change of pillar industries and sketch the industrialization process of Chinese cities in past decades.

Third, this study focused on the emission reduction potentials of key industrial sectors only, which may skew the results toward cities with more industry and power generation. We paid less attention to the cities’ nonindustrial sectors (farming, construction, services, and households) and their emission-saving potentials. This was primarily restricted by the emission data for nonindustrial sectors. Further studies should try to improve estimation of emissions in urban service sectors. We would like to conduct a large survey to measure the differences between emission intensities of service-related sectors across 50+ selected cities in China.

## Supplementary Material

http://advances.sciencemag.org/cgi/content/full/4/6/eaaq0390/DC1

## SUPPLEMENTARY MATERIALS

Supplementary material for this article is available at http://advances.sciencemag.org/cgi/content/full/4/6/eaaq0390/DC1 fig. S1. Energy and sector mix of 182 cities’ CO_2_ emissions. fig. S2. Raw coal moved between China’s 10 largest coal-producing regions and the 10 regions with the greatest coal consumption regions in 2010. fig. S3. Sector-specific emission reductions under three scenarios. fig. S4. City spatial distribution and its corresponding provinces. table S1. Emissions and socioeconomic index of 182 Chinese cities in 2010. table S2. Sectoral categories by the Chinese National Administration for Quality Supervision and Inspection and Quarantine. table S3. Fossil fuel types and emission factors used in this study. table S4. Industry processes involved in this study and related emission factors. table S5. Energy balance tables in China energy statistical system. data S1. One hundred eighty-two city inventories. References (*66*, *67*)
