# Supplementary material for: City-level climate change mitigation in China
Source: Sci Adv. 2018 Jun 27;4(6):eaaq0390. doi: 10.1126/sciadv.aaq0390 (PMC6021142; doi:10.1126/sciadv.aaq0390)
Supplement: http://advances.sciencemag.org/cgi/content/full/4/6/eaaq0390/DC1 [file supp_4_6_eaaq0390__index.html]

Science Advances | Science Advances

## Supplementary Materials

**This PDF file includes:**

- fig. S1. Energy and sector mix of 182 cities' CO2 emissions.
- fig. S2. Raw coal moved between China's 10 largest coal-producing regions and the 10 regions with the greatest coal consumption regions in 2010.
- fig. S3. Sector-specific emission reductions under three scenarios.
- fig. S4. City spatial distribution and its corresponding provinces.
- References (*66, 67*)

Download PDF

**Other Supplementary Material for this manuscript includes the following:**

- table S1 (Microsoft Excel format). Emissions and socioeconomic index of 182 Chinese cities in 2010.
- table S2 (Microsoft Excel format). Sectoral categories by the Chinese National Administration for Quality Supervision and Inspection and Quarantine.
- table S3 (Microsoft Excel format). Fossil fuel types and emission factors used in this study.
- table S4 (Microsoft Excel format). Industry processes involved in this study and related emission factors.
- table S5 (Microsoft Excel format). Energy balance tables in China energy statistical system.
- data S1 (Microsoft Excel format). One hundred eighty-two city inventories.

Download Tables S1 to S5

**Files in this Data Supplement:**

- Adobe PDF - aaq0390\_SM.pdf
