# Supplementary material for: City-level climate change mitigation in China
Source: Sci Adv. 2018 Jun 27;4(6):eaaq0390. doi: 10.1126/sciadv.aaq0390 (PMC6021142; doi:10.1126/sciadv.aaq0390)
Supplement: http://advances.sciencemag.org/cgi/content/full/4/6/eaaq0390/DC1 [file aaq0390_SM.pdf]

Science Advances

AAAS

advances.sciencemag.org/cgi/content/full/4/6/eaaq0390/DC1

Supplementary Materials for

City-level climate change mitigation in China

Yuli Shan, Dabo Guan, Klaus Hubacek, Bo Zheng, Steven J. Davis, Lichao Jia, Jianghua Liu, Zhu Liu, Neil Fromer, Zhifu Mi, Jing Meng, Xiangzheng Deng, Yuan Li, Jintai Lin, Heike Schroeder, Helga Weisz, Hans Joachim Schellnhuber

Published 25 June 2018, *Sci. Adv.* **4**, eaaq0390 (2018)
DOI: 10.1126/sciadv.aaq0390

The PDF file includes:

- fig. S1. Energy and sector mix of 182 cities' CO_2 emissions.
- fig. S2. Raw coal moved between China's 10 largest coal-producing regions and the 10 regions with the greatest coal consumption regions in 2010.
- fig. S3. Sector-specific emission reductions under three scenarios.
- fig. S4. City spatial distribution and its corresponding provinces.
- References (*66*, *67*)

Other Supplementary Material for this manuscript includes the following:
(available at advances.sciencemag.org/cgi/content/full/4/6/eaaq0390/DC1)

- table S1 (Microsoft Excel format). Emissions and socioeconomic index of 182 Chinese cities in 2010.
- table S2 (Microsoft Excel format). Sectoral categories by the Chinese National Administration for Quality Supervision and Inspection and Quarantine.
- table S3 (Microsoft Excel format). Fossil fuel types and emission factors used in this study.
- table S4 (Microsoft Excel format). Industry processes involved in this study and related emission factors.
- table S5 (Microsoft Excel format). Energy balance tables in China energy statistical system.
- data S1 (Microsoft Excel format). One hundred eighty-two city inventories.

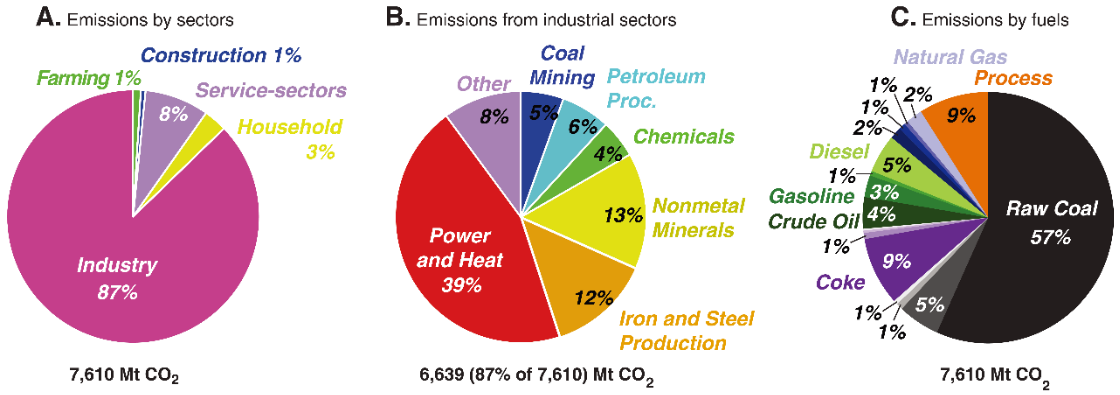

fig. S1. Energy and sector mix of 182 cities' CO2 emissions.

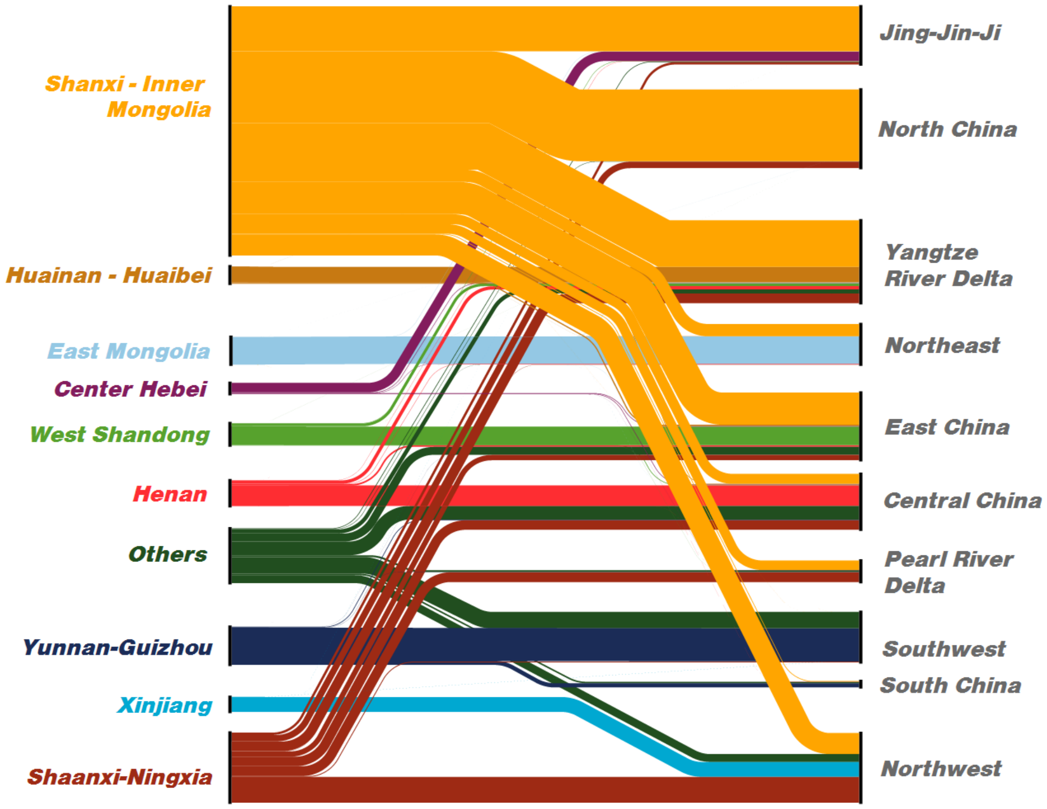

fig. S2. Raw coal moved between China's 10 largest coal-producing regions and the 10 regions with the greatest coal consumption regions in 2010. Based on the "proximity principle" in energy usage, fossil fuels are usually consumed in cities near the source area, rather than delivered to distant cities in order to reduce the overall transportation cost. For example, raw coal produced in Huainan-Huaibei coal base, which is located in Anhui, are mostly used in Yangtze River Delta. Nearly all the coal used in Jing-Jin-Ji region and north China are produced in the nearby Shanxi-Inner Mongolia coal base. Note that the Yangtze river delta includes Anhui province and the Pearl river delta refers to Guangdong province [Data source: China coal industry yearbook 2011 (*66*, *67*)].

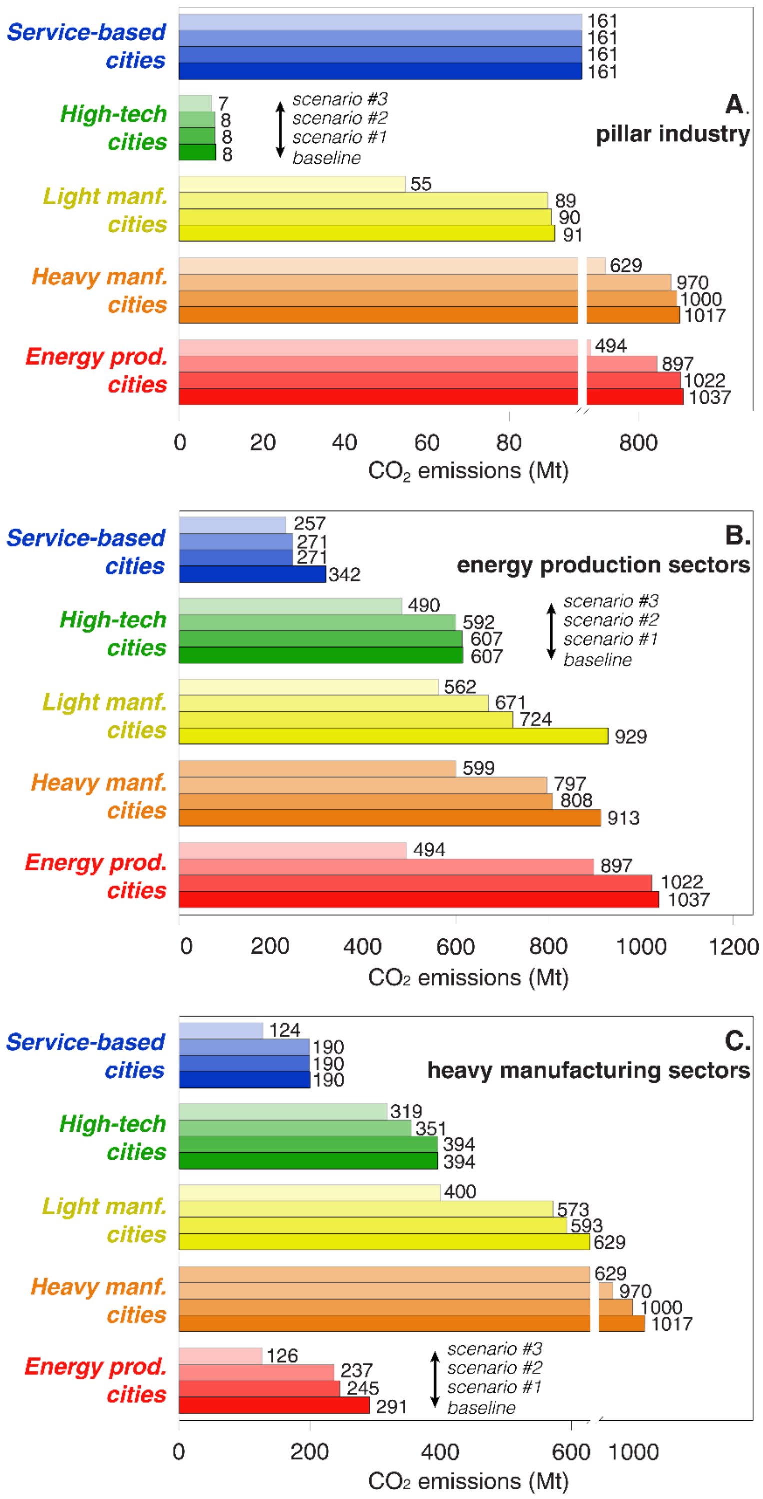

fig. S3. Sector-specific emission reductions under three scenarios. Potential reductions in CO_2 emissions for each of the five city groups where the emissions intensities of '2 s.d.', '1 s.d.', and 'above-average super-emitters are brought down to the sector mean intensity (scenarios #1, #2, and #3, respectively).

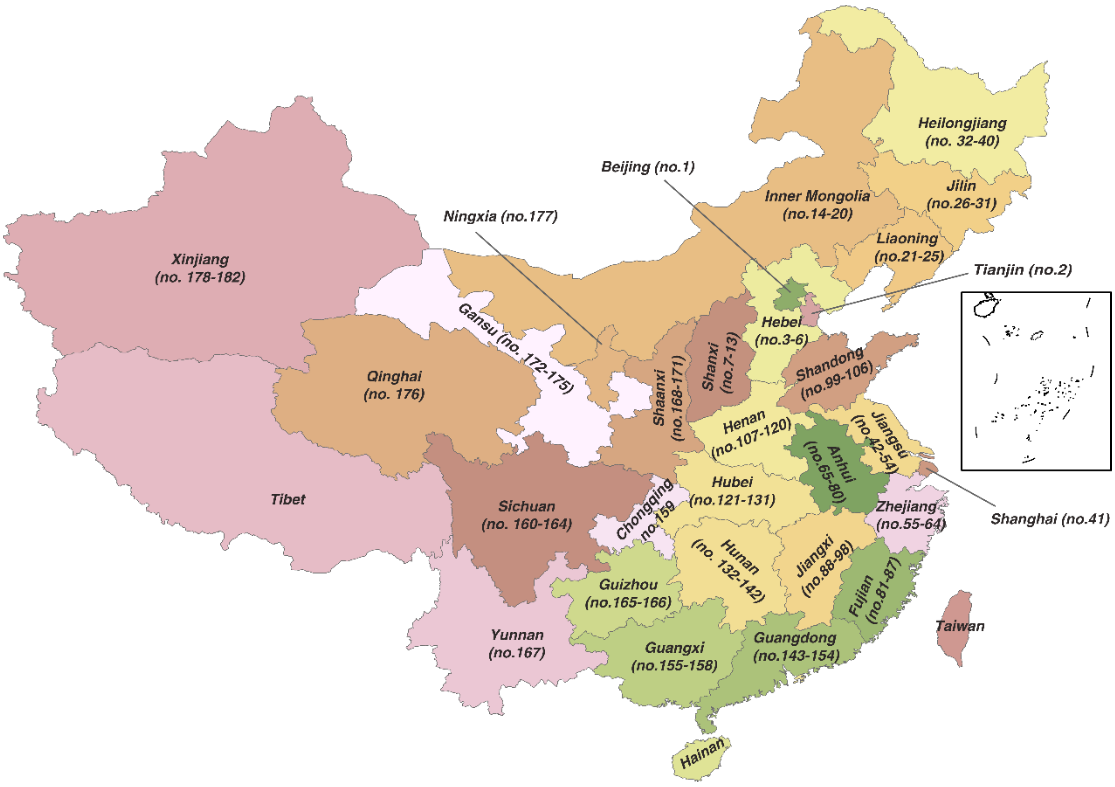

fig. S4. City spatial distribution and its corresponding provinces. The numbers in the brackets represent the cities, which are the same with city number in table S1.
